# Startling similarity: Effects of facial self-resemblance and familiarity on the processing of emotional faces

**DOI:** 10.1371/journal.pone.0189028

**Published:** 2017-12-07

**Authors:** Johannes B. Finke, Mauro F. Larra, Martina U. Merz, Hartmut Schächinger

**Affiliations:** Institute of Psychobiology, Department of Clinical Psychophysiology, University of Trier, Trier, Germany; University of Montreal, CANADA

## Abstract

Facial self-resemblance has been associated with positive emotional evaluations, but this effect may be biased by self-face familiarity. Here we report two experiments utilizing startle modulation to investigate how the processing of facial expressions of emotion is affected by subtle resemblance to the self as well as to familiar faces. Participants of the first experiment (I) (*N* = 39) were presented with morphed faces showing happy, neutral, and fearful expressions which were manipulated to resemble either their own or unknown faces. At SOAs of either 300 ms or 3500–4500 ms after picture onset, startle responses were elicited by binaural bursts of white noise (50 ms, 105 dB), and recorded at the orbicularis oculi via EMG. Manual reaction time was measured in a simple emotion discrimination paradigm. Pictures preceding noise bursts by short SOA inhibited startle (prepulse inhibition, PPI). Both affective modulation and PPI of startle in response to emotional faces was altered by physical similarity to the self. As indexed both by relative facilitation of startle and faster manual responses, self-resemblance apparently induced deeper processing of facial affect, particularly in happy faces. Experiment II (*N* = 54) produced similar findings using morphs of famous faces, yet showed no impact of mere familiarity on PPI effects (or response time, either). The results are discussed with respect to differential (presumably pre-attentive) effects of self-specific vs. familiar information in face processing.

## Introduction

Most people are inclined to see others similar in physical appearance to themselves as more trustworthy and likable [1]. This bias toward self-resembling faces may even translate to more favorable prosocial attributions and higher willingness to engage in mutual cooperation [2, 3] (but see [4] for diverging evidence). From an evolutionary point of view, facial self-resemblance has therefore been ascribed a role as a potential cue for kin recognition, evolved to prevent inbreeding and enhance cooperation among genetic relatives (by means of phenotype matching). However, favorable evaluation of similarity to the self is also consistent with the general positivity bias linked to the processing of self-related information, both at an explicit [5] and implicit level [6], as well as with the even more basic preference for familiarity (i.e., the ‘mere exposure’ effect [7]). In line with this, self-enhancement has also been observed in self-face recognition, under conditions of both supraliminal [8] and subliminal presentation [9], and also with faces bearing only partial resemblance to the self. For example, pictures of the self-face morphed with trustworthy looking individuals have been repeatedly found to be more readily identified as ‘self’ than their less trustworthy [10] or less attractive [11] counterparts. Moreover, trustworthy behavior in itself appears to inspire subjective attributions of physical self-resemblance [12]. Overall, these findings seem to suggest a connection between self-resemblance and positive emotion. However, despite initial evidence that recognition of facial identity (i.e., familiarity) and features related to emotional expressions may interact during the processing of emotional faces [13–15], contrary to previous models of face perception [16], little is known about a potential interplay of self-resemblance and emotion processing. Given that a growing body of research has documented various effects of self-resemblance on subjective evaluations of both adult [17] and child faces [18], as well as on physiological reactions to appetitive stimuli such as erotic nudes [19], the present study aimed at exploring how self-resemblance might affect responses to facial expressions of emotion. So far, even though previous studies have proposed a close relationship between representations of the self-face, on the one hand, and emotion recognition [20] as well as social cognition [21], on the other, research on the modulation of the processing of facial affect by partial similarity to the self is missing.

As predicted by appraisal theories of emotion, certain facial expressions (e.g., anger and fear) may be perceived as inherently ambiguous, relying on further contextual cues [22], such as gaze direction. Being one of the most salient and basic cues of self-relevance, direct gaze has been shown to modulate spontaneous mimic and autonomic reactions to emotional faces [23], corresponding patterns of Amygdala activation [24], as well as appetitive responses to erotic nudes [25]. However, even more complex features, such as the expresser’s in-group vs. out-group status [26], may contribute to processes underlying emotional appraisal. In line with this, there is also evidence for a reciprocal association between (positive) emotion and the perception of familiarity, which might enhance the processing of happy faces, and vice versa, resulting in higher subjective valence and arousal [14] and better discrimination performance [15]; at the same time, faces showing happy expressions are more likely to be rated as familiar [27].

Since partial self-resemblance represents both a case of subtle familiarity and an indirect cue of relevance to the self, we wondered whether it might also be able to influence spontaneous reactions to emotional faces, even when processed without conscious awareness. Indirectly, this idea is supported by recent evidence that multisensory stimulation which can induce a heightened sense of physical similarity to the self [28, 29] also facilitates recognition of fearful emotional expressions in others [30], in line with the notion that mapping between self and other might play a fundamental role in the processing of facial expressions [31]. Consistent with this, self-resemblance has already been found to boost the gaze-cueing effect [32], i.e., the modulation of automatic attentional capture by facial gaze direction. Moreover, as reported by [20], preceding presentation of one’s own face improved emotion recognition performance with respect to dynamically changing facial expressions, especially in participants scoring high in autistic traits. The apparent overlap of the human mirror neuron system and the neural substrates underlying self-recognition [21] points to changes in activation of corresponding sensorimotor nodes as a potential cause of such enhanced recognition of eye gaze and facial expression (induced by either the self-face or faces perceived as similar to one’s own). In fact, recognizing emotional states in others might be largely based on mapping them to the representation of one’s own body [33], which perhaps even forms the affective basis of empathy [34].

While implicit reference to the self has been associated with deeper emotional processing in other domains (e.g., with verbal materials; as indexed by event-related potentials and enhanced memory performance [35, 36]), face recognition is undoubtedly particularly interesting in terms of self-other discrimination, since faces convey simultaneous information about both affect and identity. Given the high extent of automaticity ascribed to both face perception in general [37, 38], and self-face recognition in particular [39, 40], a rapid interplay of self-relatedness and emotional expression at early stages of face processing could be expected. However, since the encoding of invariant structural features, underlying recognition of familiar faces, is thought to rely on neural substrates largely different from those responsible for emotion recognition [41], such timing questions remain entirely unresolved.

Startle methodology may help. Expanding on previous findings of startle modulation by facial self-resemblance [42], the aim of the current study was to explore how and when the processing of emotional faces might be affected by subtle cues of similarity to the self, unbeknownst to the participants. To address these issues, modification of the startle eye-blink reflex is a highly suitable approach, since acoustic startle elicitation can be precisely timed with respect to concurrent presentation of visual stimuli, enabling to dissociate different stages of picture processing. When measured at long latencies (‘lead intervals’) between picture onset and subsequent reflex elicitation (in the range of several seconds), startle eye-blink magnitude (i.e., maximum EMG amplitude above baseline) is typically somewhat amplified in relation to trials without concurrent picture presentation [43]. This pattern appears to be even more pronounced when top-down attention is directed at the non-startling visual stimulus [44]. Compared to neutral pictures, however, affective startle modulation, i.e., attenuation by pleasant/appetitive stimuli and facilitation by unpleasant/aversive stimuli is usually observed [45], probably largely mediated by projections from the amygdala to the startle center in the pontine reticular formation [46, 47]. At short lead intervals (e.g., 300 ms), however, prepulse inhibition of startle (PPI) is found, considered to reflect gating mechanisms subserved by the superior colliculus via inhibitory projections from the tegmentum [48]. Differences in PPI may therefore be employed as an index of automatic attentional capture varying as a function of stimulus salience [49]. When complex pictures are used as visual prepulse stimuli, startle responses are maximally inhibited at onset delays of about 300 ms [50]. Correspondingly, the earliest differential effects of (emotional) picture content on startle magnitude have been reported for lead intervals ranging from 250 ms to 300 ms [51–53].

In the present study, mean proportional change in startle magnitude (for reasons of brevity referred to as %PPI/PPF, i.e., prepulse inhibition/facilitation) was employed as an index of stimulus-induced changes in startle reactivity, which has been established as a highly robust and reliable measure across various stimulus conditions [54]. Based on highly automatic processes, startle modulation has the additional advantage of being much less prone to response biases and demand characteristics than conventional measures such as rating scales, questionnaires, and even reaction times, and is sensitive to differences in facial expression as well [55, 56]. There is also initial evidence for differential effects of emotional faces on startle at various points during the processing stream [57]. In addition to startle modulation assessed at both short and long lead intervals, we measured manual response time in a simple emotion discrimination paradigm in order to ensure proper encoding of faces and to provide converging evidence that similarity to the self-face may influence the efficiency with which emotional expressions are processed. Finally, all pictures were subsequently rated for subjective levels of valence and arousal. Besides neutral faces, we chose to include happy and fearful facial expressions (instead of angry ones, for example), because those are emotional categories most strongly associated with self-referential processing by prior research.

In line with previous results, we anticipated generally lower startle responses in short lead interval trials (i.e., PPI effects, which should be particularly pronounced when emotional faces are presented). At long lead intervals, relatively higher startle reactivity was expected, especially during presentation of unfamiliar portraits showing aversive facial expressions. Given both the assumption of a ubiquitous self-positivity effect and findings of altered processing of happy expressions obtained with familiar faces, we predicted the strongest impact of self-resemblance on responses to positive facial emotion. However, this could either be reflected in enhanced attenuation of startle, in line with the motivational priming hypothesis [58], or in relatively facilitated startle responses, which have been associated with ‘deep’ (i.e., more elaborate) encoding strategies (as opposed to rather ‘shallow’, i.e., merely perceptual processing [59, 60]). Thus, if self-resemblance simply causes faces to look more pleasant or appealing, a valence-congruent overall reduction in startle magnitude seems likely. However, given that it may lead to selective increases in depth of stimulus processing, the opposite pattern of results could be expected.

Since we were interested in implicit effects of self-resemblance on affective responses, rather than conscious self-evaluation, special care was taken to conceal the manipulation from participants. Because most previous studies on related issues either employed explicit self-other discrimination tasks or did not control for potential confounds of familiarity, it cannot be excluded that some of the effects attributed to physical similarity to the self-face do in fact result from the relatively higher familiarity of self-resembling facial characteristics. This objection is also relevant in view of the fact that the putative distinctiveness of self-face recognition has been challenged by some [61] (see also [62] for a critical review of self-specialty across a broader range of domains). Therefore, after conducting a first study designed to contrast pictures of self-resembling faces with other-resembling ones (Exp. I), we tried to replicate our initial findings in a second experiment using morphs of famous faces (Exp. II). In view of the subtlety of the manipulation, we opted for addressing these issues within two separate, yet closely matched experiments, both in order to show an effect of self-resemblance in the first place, and to rule out potential carry-over effects between self-related and familiar stimuli.

## Experiment I

### Methods

#### Participants

Forty-one students (21 women; mean age: *M* = 23.1; *SD* = 3.9) from the University of Trier and the Trier University of Applied Sciences completed both parts of the present study (receiving either partial course credit or a monetary reward of 20 € for their participation). Putative startle non-responders (*N* = 1) had been screened out in advance on the basis of a short response test on session 1 (see Procedure). All participants reported normal or corrected-to-normal vision and did neither wear beards, nor facial piercings, nor glasses during the experimental procedure (which was required in order to get photographs suitable for digital morphing). Data from two participants were rejected either because of a disproportionate high amount of EMG artifacts (i.e., > 20% missing data) (*N* = 1) or due to technical failure during the experiment (*N* = 1). Further exclusion criteria were regular use of contact lenses, confirmed psychiatric and chronic somatic disorders, as well as any kind of hearing impairment, tinnitus, and hypersensitivity to sound (final sample size: *N* = 39). Participants also had to confirm that they neither smoke nor take illicit drugs or medication (except oral contraceptives) on a regular basis, and that they would abstain from drinking caffeinated or alcoholic beverages for at least 3 hours prior to the experiment. All procedures employed in the present study were in accordance with the Declaration of Helsinki and approved by the ethics committee of the Faculty 5 Empirical Social Sciences of Saarland University (proposal number 14–8 ‘The interplay of stress and processing of self-relevant information’).

#### Stimuli and apparatus

Stimulus presentation. During the experimental session, participants were seated in front of a 15-inch TFT screen (resolution: 1280 × 1024; refresh rate: 75 Hz) at a viewing distance of approximately 80 cm. Pictures of faces subtending a visual angle of about 7.2–8.3° (horizontally) × 8.3° (vertically) were shown in the center of the screen in front of a white background. Bursts of white noise (105 dB(A) SPL, instantaneous rise time, duration: 50 ms), presented binaurally via closed audiometric headphones (Holmco PD-81, Holmberg GmbH & Co. KG), served as acoustic startle probes. E-Prime 2.0 (PST Software, Inc.) was used for the presentation of written instructions and both visual and acoustic stimuli.

Materials. Pictures of self-resembling faces were created by digitally blending a photo of the participant’s own face (showing a neutral expression) with three different same-sex faces taken from the FACES database (Max Planck Institute for Human Development, Berlin [63]). Neutral, happy, and fearful versions of each original FACES picture were included. The stimulus set of another randomly paired same-sex participant formed the corresponding comparative condition. To limit the extent of global similarity among stimuli within each set, different FACES pictures (counterbalanced across participants) were used for the self- and other-resembling conditions.

After constructing morphing templates consisting of 213 anatomically defined points, composite stimuli were created, sharing 50% of both shape and color information of the participant’s face and the respective other (affective or neutral) face (using the ‘transforming’ procedure implemented in Java Psychomorph [64]). However, 65% (instead of 50%) of texture information of the original FACES pictures was retained in order to enhance features related to the emotional expression (such as laugh and frown lines etc.). Following the morphing procedure, hair and neck regions of the composite face were masked. In pictures showing facial expressions other than neutral, teeth were also removed and replaced with the size-adjusted teeth region of the corresponding FACES pictures.

In total, 9 self-resembling (3 neutral, 3 happy, 3 fearful) and 9 other-resembling faces were created for each participant.

#### Data acquisition

Electromyographic data were recorded in accord with the guidelines of the Society for Psychophysiological Research [65], using a Biopac MP150 recording system (Biopac Systems, Inc.) with 16 bit resolution and a sampling rate of 1 kHz. EMG activity of the musculus orbicularis oculi was assessed unilaterally via Kendall Healthcare H124SG Ag/AgCl electrodes (conductive area: 8 × 8 mm) placed below the left eye with an inter-electrode distance of about 1.5 cm. The raw signal was band-pass filtered between 10–500 Hz, with additional software filtering (28 Hz high-pass cutoff); it was rectified and smoothed online using a low-pass resistor-capacitor filter with a time constant of 10 ms.

Manual responses were recorded by means of a standard Windows keyboard and mouse.

#### Procedure

General procedure. Participants who responded to our announcement and complied with the major requirements for inclusion were first invited to a short ‘screening’ session. Upon arrival at the laboratory, they were informed about the main methods used in the experiment. After providing written consent, they filled in several questionnaires concerning general demographic and health-related information. Subsequently, the experimenter led them into the testing room where they performed a short computer-assisted task (fake task). Participants were instructed not to move, but to remain relaxed and leaned back in the chair during the whole procedure, while focusing on the screen. When the participant was sitting still, silently reading the instructions given on the screen, we took several pictures of the participant’s face by means of a high-resolution USB cam mounted right on top of the display, unnoticed by the participants themselves.

After completing the fake task, participants underwent a short startle response test (containing 10 acoustic startle probes). Only participants who exhibited a startle eye-blink response above baseline EMG in more than half of the testing trials, and who matched the other inclusion criteria (see above) were admitted to the second part of the study, which was scheduled about one week later. In the meantime, an individual set of morphed pictures was prepared. After finishing the startle modulation paradigm at the second appointment, participants were asked to fill in a short post-experimental questionnaire presented on the screen (including questions whether they had noticed anything special about the stimuli, to make sure that they did not recognize themselves consciously in the pictures). Then we had participants rate all pictures used in the preceding experiment. At the end of the study, participants were thanked and fully debriefed.

Startle modulation paradigm. The main part of the experiment consisted of three consecutive blocks of trials, separated by recovery breaks of 2 min. Participants were told to relax, not to move, and to focus on the series of pictures presented on the screen. In order to familiarize them with the experimental task, 12 practice trials which involved the presentation of two additional faces (varying in emotional expression) were performed at the start of the session. After that, before continuing with the experiment, 6 startle probes were administered to allow for initial habituation of the startle response. Likewise, the second and third blocks were preceded by three of such habituation trials. Additional startle probes (serving as a reference for calculation of %PPI/PPF scores) were presented during the inter-trial period (in 6 randomly chosen trials per block). All blocks consisted of 54 trials presented in fully randomized order. In every block, each single face picture was shown three times, once in the short lead interval condition, once in the long lead interval condition, and once without concurrent presentation of a startle-eliciting stimulus. After completing the startle modulation paradigm and the post-experimental questionnaire, all pictures were shown again for a duration of 4 s each and rated for subjective valence and arousal using a visual analogue scale.

**Fig 1:**
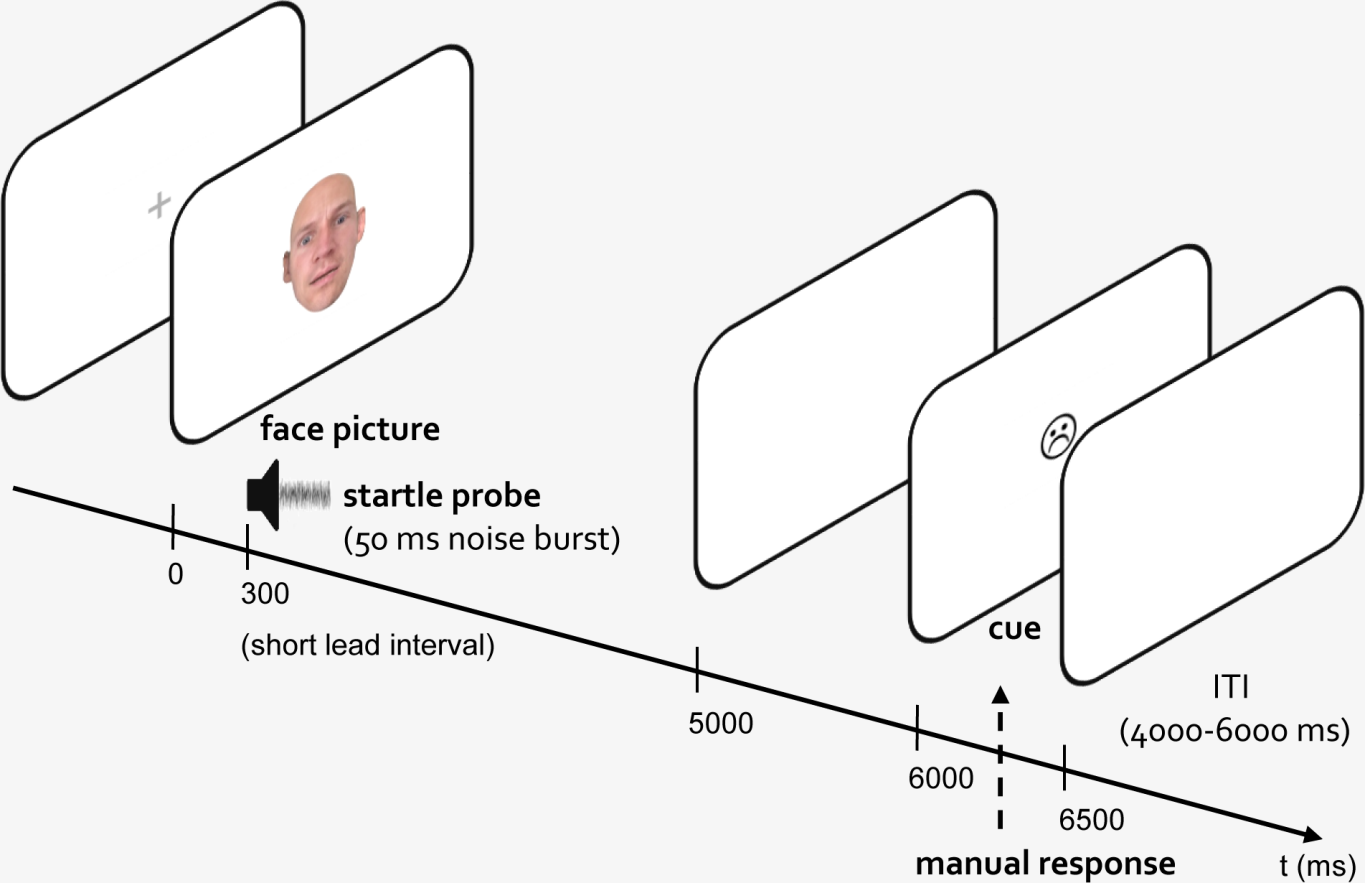
Structure of an example trial. After a delay of either 300 ms (short lead interval) or 3500–4500 ms (long lead interval) relative to picture onset, startle was elicited by a 50-ms burst of white noise (startle probe). In one third of trials no startle probe was presented. After picture offset, participants had to respond to a smiley symbol (response cue) by indicating a match or mismatch to the expression of the preceding face picture. ITI: Inter-trial interval. (The individuals whose photographs were used for the morphed image shown in this picture have given written informed consent, as outlined in PLOS consent form, to publish these details).

The structure of trials was as follows: Immediately after presentation of a fixation cross (shown for an average duration of 1500 ms, ranging from 1250 to 1750 ms), a face picture was shown for 5000 ms. Startle was elicited by acoustic probes presented at a SOA of either 300 ms after picture onset (short lead interval) or after an interval varying randomly between 3500 and 4500 ms (long lead interval); picture presentation was followed by a blank screen for 1000 ms. Subsequently, a smiley symbol showing either a positive, negative, or neutral expression appeared in the center of the screen (for 500 ms). A blank screen was then shown for an inter-trial interval (ITI) jittering randomly from 4000 to 6000 ms. See Fig 1 for an illustration of an example trial.

Participants had to indicate by pressing one of two buttons (either with their left or right hand) whether the expression of the target/response cue (i.e., the ‘smiley’) matched the expression of the preceding face (or not). Button assignment was counterbalanced across participants. No feedback on performance was given (except during practice trials).

#### Data reduction and analysis

Startle eye-blink magnitude. EMG data were analyzed by means of a C++ based customized program designed to identify response peaks in the rectified and integrated EMG signal during a time interval of 20 to 150 ms after startle probe onset. In addition, all responses were inspected manually to check for both electrical and physiological artifacts. Trials contaminated with artifacts (e.g., immediately preceded by spontaneous blinks) were rejected from analysis and defined as missing (10.6% on average across the sample; *SD* = 5.1). If there was no visible response at the typical response latency of a particular participant, response amplitude was set to zero. Startle magnitude was defined as the difference between EMG peak and baseline amplitude for each trial (with baseline set to the average value across an artifact-free interval about 50 ms prior to startle probe presentation). Zero responses (1.9% on average; *SD* = 4.5) were included in further analyses [65]. Startle responses elicited during ITI periods were used to calculate proportional change scores, indicating relative levels of startle inhibition (i.e., percentage PPI) or facilitation (i.e., percentage PPF), respectively [54]. This was done by subtracting mean startle magnitude on ITI control trials (averaged over 6 trials per block) from each response value, divided by mean ITI magnitude, i.e., by applying the formula:

$$\%PPI/PPF=\;\left(magnitude_{response}-mean\;magnitude_{ITI}\right)/mean\;magnitude_{ITI}*100$$

Reaction times (RT). Before aggregating RTs based on accurate responses (95.8% on average; *SD* = 3.7), trials with extremely fast (physiologically implausible RTs below 100 ms) as well as very slow reactions (above 1500 ms) were regarded as outliers and discarded. Moreover, to avoid potential confounding effects of startle on RT performance, due to distraction or ‘processing interrupt’ [59] effects on picture processing, only startle-free trials (i.e., trials without concurrent presentation of picture and startle-eliciting stimulus (one third of trials) were included in further analyses.

Subjective ratings. Participants had to indicate their ratings of arousal and valence by means of a computer mouse, using a visual analogue scale shown on the screen. To ensure the validity and comparability of subjective judgments, non-verbal anchor symbols, i.e., Manikins adapted from [66], were presented above the scale. The order of presentation of different rating categories was randomized. Distances between pixels were converted into arbitrary units ranging from 0 to 100.

Statistical analyses. First, data from each outcome measure were subjected to separate repeated-measures ANOVAs involving EXPRESSION (*fearful*, *neutral*, *happy*) and Similarity (*self*, *other*) as within-subjects variables. Compatibility (*matching*, *non-matching*) was included as an additional factor in RT analysis, based on the assumption that slower responses to non-matching pairs of subsequent stimuli (face picture–target) should reflect interference due to incongruent emotional content. Significant effects involving Expression were followed by sets of planned polynomial contrasts. To dissociate (significant) interactions with Similarity further, differences between responses to *self-* vs. *other-*related pictures were compared by means of simple effects analyses.

All statistical analyses were calculated using SPSS 22 (IBM SPSS Statistics) with critical α-level set to *α* = .05. A Huynh-Feldt correction of degrees of freedom was applied whenever appropriate. In that case, the applied epsilon (HF-*ε*) is reported along with the uncorrected degrees of freedom. If not stated otherwise, *p*-values of follow-up tests are given as two-tailed.

### Results

#### Startle eye-blink modulation

**Fig 2:**
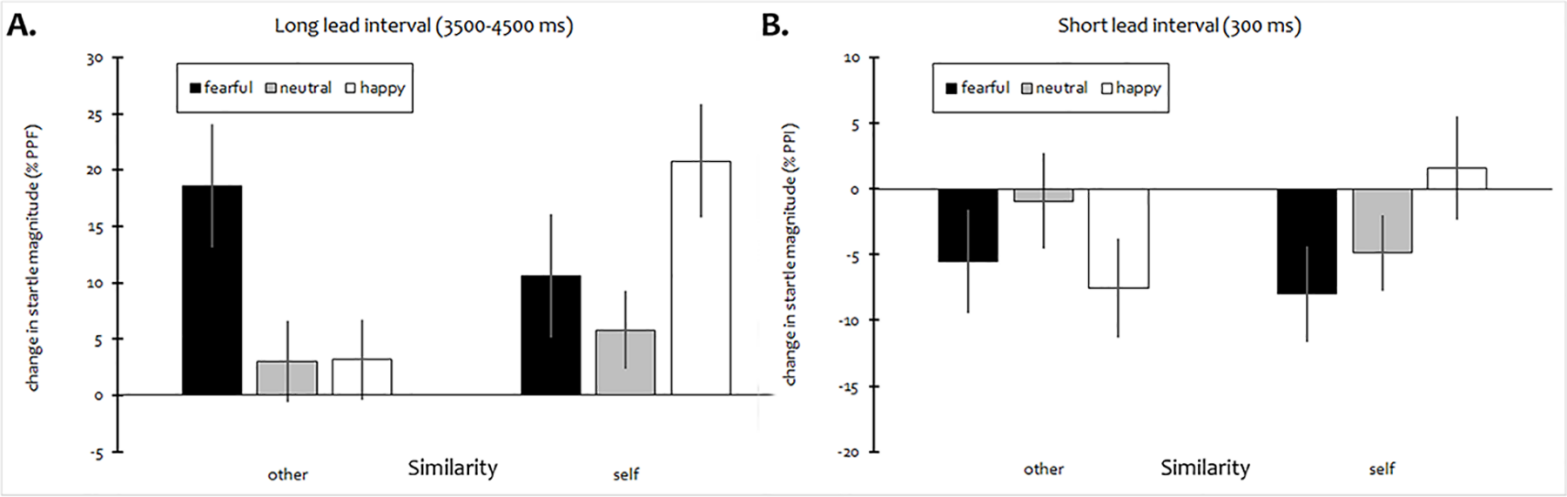
Mean proportional change in startle magnitude during presentation of morphed faces with different emotional expressions as a function of facial resemblance (self vs. other). A. long lead interval (3500–4500 ms, PPF), B. short lead interval (300 ms, PPI). Error bars represent SEM.

Long lead interval (3500–4500 ms). The overall ANOVA of mean changes in startle reactivity at long lead intervals yielded a significant main effect of Expression, *F*(2,76) = 3.57, *p* = .033, *η*_*p*_^2^ = 0.086, which was modified by a significant Expression × Similarity interaction, *F*(2,76) = 4.64, *p* = .022 (HF-*ε* = .747), *η*_*p*_^2^ = 0.109, indicating different patterns of startle facilitation by facial expressions in self- vs. other-resembling faces (mean %PPF/PPI values for each condition are given in Fig 2). The main effect of Similarity was only marginal, *F*(1,38) = 3.41, *p* = .073, *η*_*p*_^2^ = 0.082. Separate sets of planned polynomial contrasts computed for each level of Similarity showed a significant negative linear trend for emotional faces morphed with another person’s face, *F*(1,38) = 7.91, *p* = .008, *η*_*p*_^2^ = 0.172 (with marginal contribution of a quadratic effect, *F*(1,38) = 2.83, *p* = .10, *η*_*p*_^2^ = 0.069). By contrast, the pattern of responses to self-resembling faces was best approximated by a (positive) quadratic trend, *F*(1,38) = 6.75, *p* = .013, *η*_*p*_^2^ = 0.151. As indicated by analysis of simple effects, facilitation of startle was relatively more pronounced during presentation of self-resembling happy faces (*p* < .001), whereas no significant differences between *self* and *other* emerged with other facial expressions (all *p*s > .25).

In the other-resembling control condition, facilitation of startle (significant differences from zero, one-tailed) was observed with fearful faces only (*p* = .001). In line with the results described above, this pattern was substantially changed by the presence of resemblance to one’s own face, which induced significant PPF both with positive (*p* < .001) and (*p* = .032) negative facial expressions.

Short lead interval (300 ms). Prepulse inhibition of startle elicited at short lead intervals (see Fig 2) was modulated by an interaction of Expression and Similarity, *F*(2,76) = 4.10, *p* = .020, *η*_*p*_^2^ = 0.097, while both main effects did not approach significance (all *F*s < 1.4, *p*s > .25). Specifically, the other-resembling control condition was marked by a negative quadratic effect (even though significant only on a trend level: *F*(1,38) = 3.41, *p* = .073, *η*_*p*_^2^ = 0.082), as expected for the contrast between emotional and neutral facial expressions, whereas startle inhibition in response to self-resembling faces linearly decreased with emotional valence, *F*(1,38) = 6.92, *p* = .012, *η*_*p*_^2^ = 0.154. Simple effects analyses corroborated that startle responses were less attenuated during presentation of self-resembling happy faces, as compared to other-resembling ones (*p* = .014), similar to the pattern of results observed at long lead intervals.

Significant PPI (*p*s < .05, one-tailed) was observed in all stimulus conditions except *fearful*/*other*, *neutral*/*other*, as well as *happy*/*self*.

#### Reaction time (emotion discrimination)

The overall ANOVA performed on the basis of mean RT data revealed a significant main effect of Compatibility, *F*(1,38) = 10.41, *p* = .003, *η*_*p*_^2^ = 0.215), indicating overall faster responses to matching as compared to non-matching targets. Moreover, there was also a main effect of Expression, *F*(2,76) = 30.41, *p* < .001, *η*_*p*_^2^ = 0.445, which was qualified by a significant Expression × Compatibility interaction, *F*(2,76) = 7.83, *p* = .002 (HF-*ε* = .822), *η*_*p*_^2^ = 0.171. Importantly, also a significant main effect of Similarity emerged, *F*(1,38) = 6.83, *p* = .013, *η*_*p*_^2^ = 0.152. No other two- or three-way interactions attained significance (*F*s < 1.6, *p*s > .2).

As indicated by planned polynomial contrasts, both a pronounced linear trend as a function of facial Expression (faster responses with rising levels of picture valence, from negative to positive; *F*(1,38) = 56.20, *p* < .001, *η*_*p*_^2^ = 0.597) and a linear increase in the effect of Compatibility emerged, *F*(1,38) = 13.96, *p* < .001, *η*_*p*_^2^ = 0.269, both pointing toward more efficient processing with rising levels of picture valence. Follow-up comparisons further confirmed that participants reacted faster to matching (than non-matching) targets after presentation of happy (*p* < .001) as well as neutral faces (*p* = .023), but not after viewing of fearful ones (*p* > .5).

**Fig 3:**
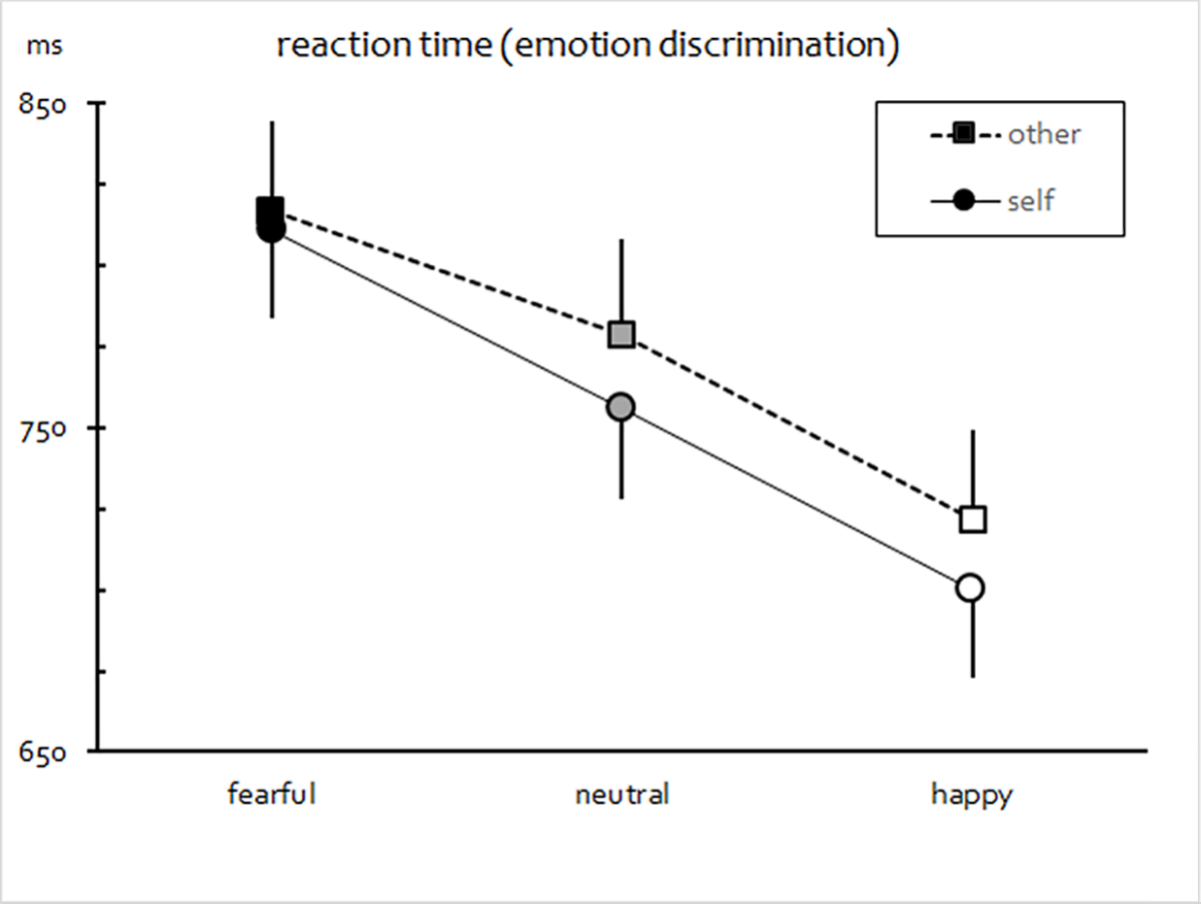
Mean RT as a function of emotional expression and facial resemblance (self vs. other). Error bars represent SEM.

Most importantly, however, reaction time was also modulated by facial resemblance, with faster average responses (*p* = .013) on trials when self-resembling faces had been presented. Collapsing across levels of Compatibility, Fig 3 illustrates this result.

#### Subjective ratings

Arousal. As expected, a significant main effect of Expression on ratings of subjective level of arousal was found, *F*(2,76) = 56.01, *p* < .001, *η*_*p*_^2^ = 0.596 (see Table 1 for means of all conditions). There was neither a main effect of Similarity nor an interaction (both *F*s < 1, *p*s > .8). Planned contrasts showed a significant quadratic trend, *F*(1,38) = 67.89, *p* < .001, but also a significant difference between fearful and happy faces, *F*(1,38) = 23.65, *p* < .001, with the latter being rated relatively lower in arousal.

**Table 1:** Mean ratings of arousal (Exp. I).

Expression	Similarity		
	*other*	*self*	Δ
**fearful**	62.8 ± 3.1	63.0 ± 3.1	-0.2
**neutral**	28.3 ± 2.7	28.8 ± 2.5	-0.6
**happy**	51.2 ± 2.8	51.2 ± 2.7	0.0

*Note*. Data are based on a visual analogue scale (ranging from 0–100) and given as mean ± standard error of the mean. In addition, difference scores (*Δ*) between other- vs. self-resembling stimuli have been included.

Valence. In the analysis of judgments of picture valence (see Table 2) only a significant main effect of Expression emerged, *F*(2,76) = 278.88, *p* < .001, *η*_*p*_^2^ = 0.880. There was neither a significant main effect of Similarity, *F*(1,38) = 1.42, *p* = .241, nor an interaction (*F* < 1). Ratings of facial affect were best approximated by a linear trend, *F*(1,38) = 346.27, *p* < .001, irrespective of resemblance.

**Table 2:** Mean ratings of valence (Exp. I).

Expression	Similarity		
	*other*	*self*	Δ
**fearful**	21.1 ± 1.9	22.4 ± 1.9	-1.4
**neutral**	44.9 ± 1.2	45.0 ± 1.3	-0.1
**happy**	74.7 ± 1.9	76.9 ± 1.8	-2.2

*Note*. Data are based on a visual analogue scale (ranging from 0–100) and given as mean ± standard error of the mean. In addition, difference scores (*Δ*) between other- vs. self-resembling stimuli have been included.

Subjective awareness. In the answers to the questionnaire presented after the main part of the experiment, no participant indicated any kind of awareness regarding the true nature of the experimental manipulation, i.e., neither did anyone make reference to recognizing his/her face (or any kind of resemblance), nor did any participant correctly guess any of the hypotheses underlying our study.

### Discussion

Both explicit and implicit reference to the self has been shown to modulate emotional responses [36], particularly with regard to positively valenced stimuli, i.e., in a self-image congruent way [35]. Moreover, the representation of one’s own face might play a crucial role in social cognition [21, 32] and emotion recognition [20, 30]. Given this link between emotion and the bodily self, we expected the processing of emotional expressions to be influenced by similarity to the self-face. The findings of Exp. I confirm this general assumption, suggesting that subtle cues of self-resemblance are indeed associated with altered processing of facial affect, as evident both from modulation of largely automatic reactions and from indices of more controlled behavioral responses. Notably, these effects were independent of conscious recognition.

The pattern of startle modification observed with other-resembling faces was largely consistent with the standard motivational priming account [58], indicating potentiated startle responses with fearful faces, i.e., affective modulation of startle, at long lead intervals, and higher levels of startle inhibition in response to emotional faces (irrespective of valence) after very short onset delays. (Note that most studies available in the literature on affective startle modulation by facial expressions have not reported reliable differences in startle reactivity to happy faces, in relation to neutral stimuli; e.g. [55].) By contrast, viewing of self-resembling faces, as compared to morphed faces unrelated to the self, caused changes both in affective startle modulation and in pre-attentive stimulus processing, as indexed by differential, emotion-specific PPI effects at short latencies relative to picture onset. Overall, the pattern of startle results suggests that self-resemblance primarily affected automatic processing of positive facial emotion, whereas performance enhancement regarding manual responses was rather non-specific as to the emotional category. While the former finding (i.e., the modulation of automatic responses) is in line with research showing an association of facial familiarity and happy expressions [13, 14, 27], we did not find reliable evidence for comparable valence-dependent effects on manual response times. However, this is most likely due to the nature of the task employed in the current study which (unlike in some previous studies) was not based on speeded, but rather delayed categorization, requiring participants to encode and retain each emotional expression for a short time span. Moreover, even though there was no significant interaction of similarity and emotional expression, inspection of the cell means strongly suggests that the effect of similarity was mainly driven by the pronounced reduction in mean reaction time after presentation of happy and neutral faces (yet virtually absent with fearful ones). In either case, this indicates that expressions in faces similar to one’s own might have been more deeply processed (which would also account for facilitation of startle observed with positive stimulus content, as further explained below). Our findings therefore provide additional evidence that indirect reference to the self-face can facilitate emotion discrimination. Interestingly, as evidenced by data from subjective ratings (showing no impact of self-resemblance on stimulus evaluation at all), both attentional and emotional effects of facial similarity did clearly not rely on (or converge with) conscious appraisal. Rather, our results would be in line with the assumption of a dissociation of deliberate evaluations and more implicit emotional responses to self-referential stimuli.

The direction of the effect of self-resemblance on the processing of happy faces is unlikely to be explained by a mainly valence-driven mechanism, even in view of the fact that several previous studies have also reported relative potentiation of startle not only in response to negative (angry, fearful), but also positive facial emotion [57, 67]. Rather, simultaneous processing of self-resemblance apparently counteracted affective attenuation of startle, as indexed by higher levels of startle facilitation. This pattern of findings is largely reminiscent of the ‘processing interrupt’ effect on startle magnitude [59, 60], which is thought to occur when startle elicitation interferes with more elaborate or inwardly directed stimulus processing, resulting in amplified startle responses to emotional content (irrespective of valence). This interpretation would also be compatible with the notion of an underlying self-positivity bias, which might have directed more attention to self-resembling faces with positive affect (being congruent to one’s self-image [35]), prompting an even more pronounced processing interrupt effect. In line with this explanatory hypothesis, post-hoc correlational analyses performed on data from Exp. I found overall differences between startle responses to self- and other-resembling faces (at long lead intervals) to be related to self-reported self-esteem (as measured with the Rosenberg Self-Esteem Scale [68]): *r* = .322, *p* = .048 (after exclusion of one outlier ranging more than 3 SD above sample mean). Notably, several previous studies have also associated positivity effects of the self-face [9] and partial self-resemblance [11] with either explicit or implicit measures of self-esteem.

However, the above findings do not rule out that some of the effects of facial resemblance on emotional processing might in fact result from greater familiarity of self-related facial features, rather than relying on genuine reference to the self. Moreover, whether the assumption of a categorical (rather than gradual) distinction regarding the processing of self-referential vs. familiar stimuli is empirically warranted has been contested, both in general terms [69] and with respect to self-face recognition [61]. To assess this alternative hypothesis, we conducted a second experiment (including an even larger sample of participants) to investigate effects of familiar looking emotional faces on affective modulation and prepulse inhibition of startle. This was implemented by means of morphs of famous faces (tested against unknown faces as controls). A-priori power analysis indicated that a sample size of *N* ≥ 53 would be sufficient in order to achieve a probability of 1 –*β* = .90 to detect an effect comparable in size to the smallest interaction effect involving facial resemblance as found in the present study (i.e., *η*_*p*_^2^ = 0.097, corresponding to a simple effect of *d* = 0.41).

## Experiment II

### Methods

#### Participants

A new sample of 60 students (30 women; mean age: *M* = 23.4; *SD* = 2.9) from the University of Trier and the Trier University of Applied Sciences was recruited for Exp. II (all receiving 15 € for participating). Criteria for inclusion were the same as in Exp. I. Again, data from some participants (*N* = 5) had to be discarded due to high amounts (> 20%) of missing trials in EMG recordings. Nevertheless, these participants were included in analyses of RT and rating data. One participant was excluded from the whole study after mentioning upon debriefing that she had also taken part in the previous experiment. Final sample size used for analyses of startle data was *N* = 54.

#### Design, procedure, and analysis

Design, procedure, and materials, as well as data acquisition and scoring, were exactly identical to Exp. I, apart from the fact that morphs of famous faces were used instead of self-resembling stimuli. Moreover, since no pre-test session was required for the purpose of acquiring facial portraits, participants underwent the startle modulation paradigm immediately after providing questionnaire data. Each participant was randomly assigned to one out of six gender-matched sets of famous-face morphs, which had been created by utilizing photographs of German celebrities. Pictures of Lena Meyer-Landrut (singer), Heidi Klum (model/presenter), and Angelique Kerber (tennis player) were used for female participants. Matthias Schweighöfer (actor/singer), Til Schweiger (actor), and Mario Götze (soccer player) were included as male counterparts (all pictures were digitally manipulated in exactly the same manner as the morphs generated for the previous experiment). Three additional faces (per gender) unknown to the participants were employed for the other-resembling comparative condition.

Data of each dependent measure were first analyzed the same way as for Exp. I. For exploratory reasons, we conducted additional combined analyses of startle data (including Experiment as a between-subjects factor) both to corroborate convergent pattern of results and to test for specific differences between contrasts of *self* vs. *other* and *famous* vs. (*unknown*) *other*.

### Results

#### Startle eye-blink modulation

**Fig 4:**
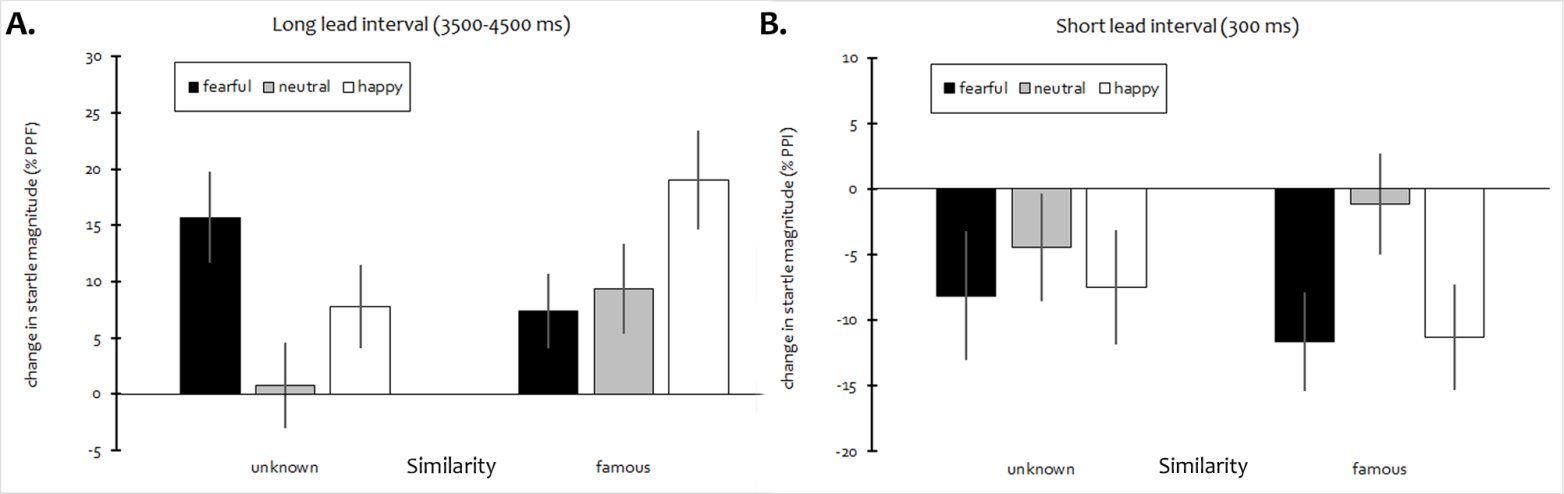
Mean proportional change in startle magnitude during presentation of morphed faces with different emotional expressions as a function of facial resemblance (famous vs. unknown). A. long lead interval (3500–4500 ms, PPF), B. short lead interval (300 ms, PPI). Error bars represent SEM.

Long lead interval (3500–4500 ms). In the analysis of startle modification by emotional morphed faces at long lead intervals there were both a significant main effect of Expression, *F*(2,106) = 3.39, *p* = .038, *η*_*p*_^2^ = 0.060, and an Expression × Similarity interaction, *F*(2,106) = 4.99, *p* = .010 (HF-*ε* = .927), *η*_*p*_^2^ = 0.086, while the effect of Similarity did not attain significance, *F*(1,53) = 2.29, *p* = .137. Planned contrasts showed a quadratic effect in the other-resembling (unknown) condition, *F*(1,53) = 9.28, *p* = .004, *η*_*p*_^2^ = 0.149, pointing to enhanced startle reactivity with emotional cues, whereas responses to famous-resembling faces increased linearly with rising valence of expression, *F*(1,53) = 5.89, *p* = .019, *η*_*p*_^2^ = 0.100 (see Fig 4). Similar to the findings observed with self-resemblance in Exp. I, simple effects analyses indicated relatively higher potentiation of startle when morphed versions of famous faces with positive expressions were presented (*p* = .029). By contrast, startle reactivity was marginally reduced with fearful morphs of famous, as compared to unknown faces (*p* = .052).

PPF of startle was significant across all various stimulus conditions (*p*s < .05, one-tailed), except for *neutral*/*unknown* faces.

Short lead interval (300 ms). In the analysis of short lead interval trials, only the main effect of Expression attained significance, *F*(2,106) = 5.82, *p* = .004 (HF-*ε* = .872), *η*_*p*_^2^ = 0.099, irrespective of facial resemblance (all other *F*s < 1.21). Startle inhibition as a function of emotional valence was best characterized by an inverse quadratic trend, *F*(1,53) = 9.76, *p* = .003, *η*_*p*_^2^ = 0.156, suggesting higher PPI in response to display of facial affect.

Correspondingly, all types of emotional expressions (collapsed across Similarity) induced significant PPI (*p*s < .01, one-tailed), yet neutral faces did not (*p* = .22).

#### Reaction time (emotion discrimination)

Due to missing values in one or more cells of the factorial design (caused by high numbers of inaccurate/late responses), 4 participants (out of 59) could not be included in RT analyses. Moreover, RT data from one participant were lost due to technical failure. Mean accuracy across the remaining sample was 93.4% (*SD*: 12.1%).

**Fig 5:**
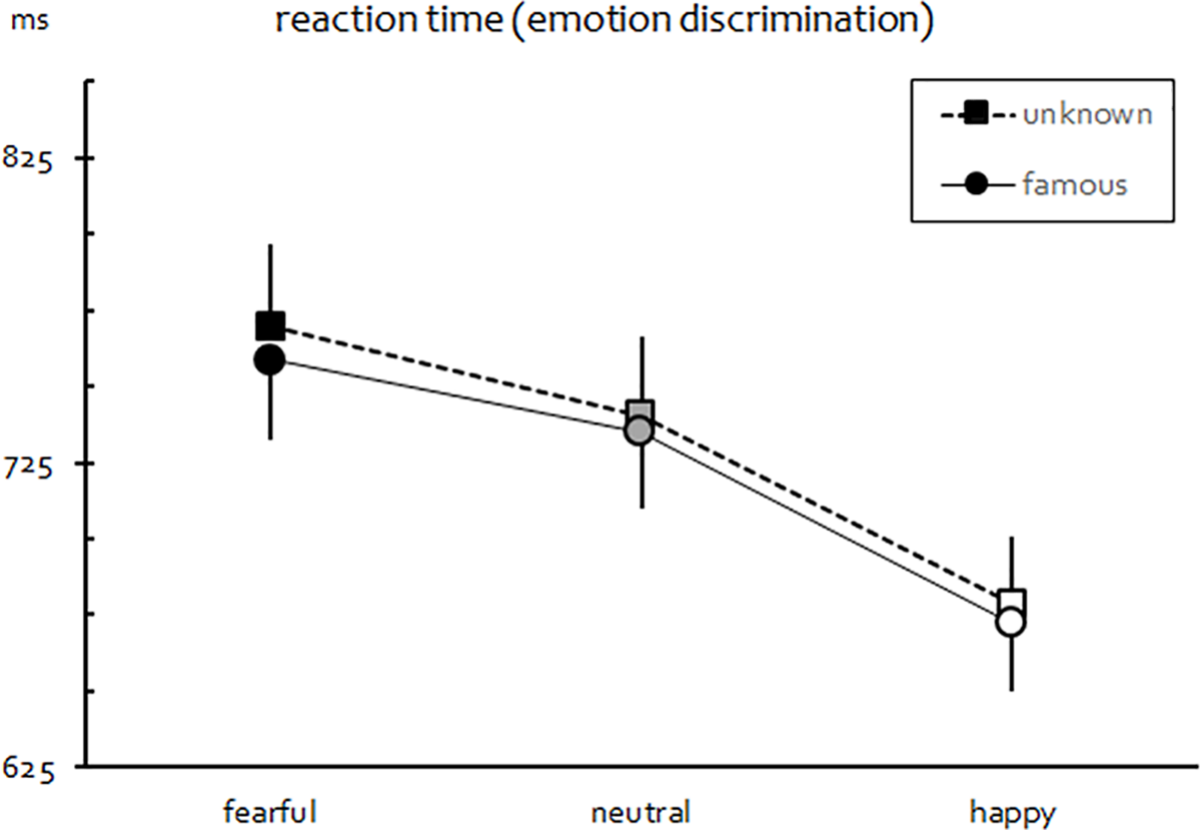
Mean RT in Exp. II. Error bars represent SEM.

As in Exp. I, significant main effects of both Compatibility, *F*(1,53) = 22.68, *p* < .001, *η*_*p*_^2^ = 0.300, and Expression, *F*(2,106) = 49.49, *p* < .001, *η*_*p*_^2^ = 0.483, on mean RTs were found. Again, a significant Expression × Compatibility interaction also emerged, *F*(2,106) = 13.78, *p* < .001, *η*_*p*_^2^ = 0.206, whereas all other effects involving Similarity did not attain significance (all *F*s < 1.1, *p*s > .29). Follow-up tests again showed the same pattern of joint effects of stimulus compatibility and positivity as previously reported. Importantly, however, there was no indication of any relevant impact of facial familiarity on response speed whatsoever (as shown in Fig 5).

#### Subjective ratings

Arousal. Means of subjective ratings of arousal (as given in Table 3) were highly similar to Exp. I. Again, a significant effect of Expression, *F*(2,116) = 79.41, *p* < .001, *η*_*p*_^2^ = 0.578 (due to a quadratic trend, *F*(1,58) = 110.41, *p* < .001, *η*_*p*_^2^ = 0.656), but no other significant effects were found (both *F*s < 1.2, *p*s > .3).

**Table 3:** Mean ratings of arousal (Exp. II).

Expression	Similarity		
	*unknown*	*famous*	Δ
**fearful**	60.2 ± 2.1	59.3 ± 2.1	1.0
**neutral**	29.4 ± 2.1	30.8 ± 2.3	-1.4
**happy**	44.2 ± 2.1	46.7 ± 2.2	-2.5

*Note*. Data are based on a visual analogue scale (ranging from 0–100) and given as mean ± standard error of the mean. In addition, difference scores (*Δ*) between unknown vs. famous-resembling stimuli have been included.

Valence. In contrast to the results obtained in Exp. I, the ANOVA performed on valence ratings of pictures used for Exp. II revealed not only the expected main effect of Expression, *F*(2,116) = 420.74, *p* < .001 (HF-*ε* = .694), *η*_*p*_^2^ = 0.879 (approximated by a linear increase, *F*(1,58) = 500.11, *p* < .001, *η*_*p*_^2^ = 0.896), but also a main effect of Similarity, *F*(1,58) = 4.11, *p* = .047, *η*_*p*_^2^ = 0.066 (whereas the interaction was clearly non-significant, *F* < 1, *p* > .6). Overall, famous-face morphs tended to be rated as slightly more pleasant as compared to unknown other-resembling faces. However, as in Exp. I, there was no sign that this minor effect of resemblance varied with facial expression (see Table 4).

**Table 4:** Mean ratings of valence (Exp. II).

Expression	Similarity		
	*unknown*	*famous*	Δ
**fearful**	25.3± 1.1	27.6 ± 1.4	-2.3
**neutral**	47.8 ± 0.7	49.4 ± 0.9	-1.6
**happy**	72.5 ± 1.5	73.4 ± 1.6	-0.9

*Note*. Data are based on a visual analogue scale (ranging from 0–100) and given as mean ± standard error of the mean. In addition, difference scores (*Δ*) between unknown vs. famous-resembling stimuli have been included.

Subjective awareness. After the main part of the experiment, none of the participants mentioned recognizing any of the faces or noticing any resemblance to existing individuals. Thus, as in Exp. I, no one reported conscious awareness of the true nature of the manipulation.

On debriefing, famous faces were judged as familiar by the participants. However, 22 participants (37.3%) were unable to freely recall the name of the famous person they had been matched with (after being presented with a picture of their face). Nonetheless, excluding these participants from statistical analyses did not affect the pattern of results in any substantial way, i.e., no additional main effect or interaction involving Similarity approached significance.

#### Combined analysis of startle data

Confirmatory analyses combining data of both experiments are shortly reported below. Regarding startle modification at long lead intervals, the overall mixed ANOVA revealed no interaction (or main effect, either) involving Experiment (all *F*s < 1), whereas both within-subjects main effects (Expression: *F*(2,182) = 6.57, *p* = .002, *η*_*p*_^2^ = 0.066, Similarity: *F*(1,91) = 5.06, *p* = .027, *η*_*p*_^2^ = 0.053) as well as their two-way interaction (*F*(2,182) = 9.12, *p* < .001, *η*_*p*_^2^ = 0.091) attained significance. Morphs of unknown faces caused significantly less facilitation of startle than the familiar (self-/famous-resembling) category only when happy faces were presented (*p* < .001). The opposite effect was found with fearful expressions, showing slightly blunted potentiation of startle due to cues of facial familiarity (*p* = .04). With neutral expressions, by contrast, there was also a trend toward higher startle responsiveness to morphs of familiar faces (*p* = .072).

Picture-induced prepulse inhibition showed a different pattern: Apart from a significant main effect of Expression (*F*(2,182) = 4.80, *p* = .009, *η*_*p*_^2^ = 0.050), only the critical three-way interaction involving Experiment and Similarity emerged (*F*(2,182) = 3.74, *p* = .029 (HF-*ε* = .923), *η*_*p*_^2^ = 0.040). As shown by direct comparisons (independent samples *t*-tests), both experimental groups differed significantly regarding the contrast between familiar and unknown faces with positive facial affect (*t*(91) = 2.68, *p* = .009), whereas no other difference approached significance (all *p*s > .16). Thus, a distinct (positivity) effect specific to processing of self-resemblance emerged at short lead intervals, while startle modulation at long lead intervals did not differ between self- and famous-resembling faces, probably relying on overall stimulus familiarity.

## General discussion

Findings from both experiments conducted for the present study show that startle reactivity during viewing of happy faces is affected by facial familiarity, being facilitated by subtle similarity both to the self and to famous faces. At long lead intervals, indexing affective modulation of startle, this pattern of results was highly comparable across both experiments reported here, clearly indicating that cues of facial identity and affect interact at later stages of face processing. However, while the finding of increased startle inhibition at short onset delays evoked by faces with emotional expressions (Exp. II) replicated the major results observed in the other-resembling condition of Exp. I, there was no effect of general facial familiarity on PPI (Exp. II); by contrast, self-resemblance (Exp. I) induced relative startle facilitation with positive emotional expressions (similar to the effects found at long lead intervals). Thus, while joint effects of facial expression and identity on startle are apparently mainly driven by overall familiarity, self-related facial features might be more easily and rapidly processed, impacting on emotion recognition at an earlier point in the processing stream. In line with this, given that several studies have linked higher depth of stimulus processing to amplified startle responsiveness specifically with positive content [60, 70], the most straightforward and parsimonious explanation for our overall findings can be based on the processing interrupt model of startle modification [59]. Consistent with both a self-enhancement bias involving one’s own face [11] and a general preference for familiarity and averageness in faces [71, 72], both self-resembling and famous-resembling faces might have been relatively more deeply processed, especially when showing happy facial expressions. This interpretation is also in agreement with electrophysiological evidence for positivity effects related to facial familiarity (shortened P300 latency following presentation of familiar happy, as compared to unhappy, faces [15]), which possibly extend to the domain of startle modulation. Alternatively, assuming that the processing of facial affect was somewhat compromised in faces bearing similarity to a known person (resulting in lower net attenuation of startle in response to happy faces) would be another potential explanation of our data. This would be compatible with the findings by [73] which indicated that facial identity can be processed without interference from emotional expression, whereas discrimination of emotion in a speeded classification task was affected by task-irrelevant variations in identity. However, analyses combining data from both experiments revealed that the effect of familiarity was apparently reversed with fearful facial expressions, which were associated with a relative reduction in startle reactivity. Notably, this pattern of results was also numerically present with self-resembling faces (Exp. I), even though not significant. Given that levels of startle facilitation during presentation of familiar faces with negative expressions were even lower than responses to happy faces, this finding renders an account simply in terms of blunted emotional responding as unlikely, pointing rather to a small, but consistent valence-dependent effect of facial familiarity on emotional responding.

Taken together, the present research suggests that facial self-resemblance might benefit from prioritized processing, as hypothesized with regard to the self-face [40, 74], and may rapidly interact with the processing of emotional cues, presumably even at an automatic, pre-attentive level, whereas effects of subtle facial familiarity appear to be restricted to later stages of face perception. This interpretation is also supported by the results found in the manual response task, which showed an impact of self-, but not famous-resemblance on discrimination performance, even though facial identity was completely task-irrelevant and manipulated without conscious awareness on the part of the participant. This result is consistent with prior studies [20], suggesting that priming with features related to the self-face can cause more efficient processing of facial expressions. Nevertheless, the available data do not permit to rule out the possibility that nonconscious perception of self-resemblance represents simply an extreme example of recognition of familiarity, rather than relying on categorically different mechanisms. Interestingly, recent research on neuropsychological disorders accompanied by feelings of hyperfamiliarity has revealed that detection of familiar faces, preceding explicit face recognition, may mainly involve right-lateralized subcortical routes, as well as areas within the right medial and inferior temporal cortex [75, 76]. As opposed to face processing in general, self-face recognition has also been associated with a right-hemispherical specialization [21, 77]. Therefore, even though there is clear evidence for a modulation of both attentional resources and affective reactions by only partial similarity to the self-face, this does not necessarily reflect effects specific to the self. However, as revealed by imaging studies, the neural substrates underlying self-recognition and detection of facial familiarity overlap to a high extent, but do also exhibit some degree of specificity (especially in frontal areas [78, 79]). Delineating intersections and dissociations of self-face recognition and the processing of familiar faces is still a promising direction for future research.

Finally, some important limitations of our study deserve to be mentioned. First, it cannot be ruled out that the morphing procedure implemented in the current study, which involved blending neutral faces with those showing emotional expressions, did somehow compromise the ecological validity of our stimuli. Nevertheless, the results obtained with the other-resembling morphs in both Exp. I and II are largely in line with predictions made on the basis of previous research (e.g., [55, 57]) and clearly confirm that both prepulse inhibition and affective modulation of startle was present. Moreover, subjective judgments of picture valence and arousal corroborate this assumption. Therefore, these findings may point to a dissociation of implicit and explicit effects in face perception and emotion recognition. In contrast to other studies using unedited pictures of personally familiar faces [14], we observed no impact of facial identity on general arousal ratings, which is probably due to the fact that only partial resemblance to familiar faces was used in the present research. Given the absence of an interaction of familiarity and expression, the overall slightly higher valence ratings of famous-face morphs (possibly resulting from higher average attractiveness) do not seem to question the comparability of both parts of our study. However, different kinds of familiarity and resemblance might exert specific and potentially diverging effects on emotional evaluation. For example, automatic evaluations of novel faces may be biased by partial similarity to faces previously linked to either affectively positive or negative impressions [80]. Famous-resembling faces might be subject to similar covert transference effects. In view of these potential confounding variables, future research should address different types of familiarity in greater detail and focus on the specific mechanisms underlying effects of resemblance on startle modulation. Likewise, the design of our study did not allow to tap into potential effects of either gender of the expresser [81] or gender of the observer [55] on startle modulation by affective faces. In addition to that, we cannot presume that all participants had exactly the same level of naivety regarding stimulus materials, even though we made sure to check for the participants’ awareness with respect to the manipulation of facial similarity.

These methodological constraints notwithstanding, the findings reported here expand on the existing literature on self-resemblance in a significant way, corroborating the notion that not only the self-face, but also faces similar to one’s own are processed in a manner different from stimuli unrelated to the self. Moreover, our study provides further evidence for an early interplay of recognition of facial identity and emotional expression in face processing, and points to interesting similarities as well as dissociations between effects of self-relatedness and familiarity.

## Supporting information

S1 File Data of both experiments. Data of startle responses (long and short lead intervals; sheet 1), subjective ratings (arousal, valence; sheet 2), and manual responses (reaction time; sheet 3); long_LI: long lead interval; short_LI: short lead interval; neg: negative; ntr: neutral; pos: positive.
(XLSX)
